# Advances in blood biomarkers for Alzheimer disease (AD): A review

**DOI:** 10.1002/kjm2.12870

**Published:** 2024-06-18

**Authors:** Araya Dimtsu Assfaw, Suzanne E Schindler, John C Morris

**Affiliations:** ^1^ Department of Neurology, Knight Alzheimer Disease Research Center (Knight ADRC) Washington University School of Medicine St. Louis Missouri USA

**Keywords:** Alzheimer disease, blood test, blood‐based biomarkers, diagnosis, plasma biomarkers

## Abstract

Alzheimer disease (AD) and Alzheimer Disease and Related Dementias (AD/ADRD) are growing public health challenges globally affecting millions of older adults, necessitating concerted efforts to advance our understanding and management of these conditions. AD is a progressive neurodegenerative disorder characterized pathologically by amyloid plaques and tau neurofibrillary tangles that are the primary cause of dementia in older individuals. Early and accurate diagnosis of AD dementia is crucial for effective intervention and treatment but has proven challenging to accomplish. Although testing for AD brain pathology with cerebrospinal fluid (CSF) or positron emission tomography (PET) has been available for over 2 decades, most patients never underwent this testing because of inaccessibility, high out‐of‐pocket costs, perceived risks, and the lack of AD‐specific treatments. However, in recent years, rapid progress has been made in developing blood biomarkers for AD/ADRD. Consequently, blood biomarkers have emerged as promising tools for non‐invasive and cost‐effective diagnosis, prognosis, and monitoring of AD progression. This review presents the evolving landscape of blood biomarkers in AD/ADRD and explores their potential applications in clinical practice for early detection, prognosis, and therapeutic interventions. It covers recent advances in blood biomarkers, including amyloid beta (Aβ) peptides, tau protein, neurofilament light chain (NfL), and glial fibrillary acidic protein (GFAP). It also discusses their diagnostic and prognostic utility while addressing associated challenges and limitations. Future research directions in this rapidly evolving field are also proposed.

## 1 INTRODUCTION

Alzheimer disease (AD) is the most common cause of dementia, affecting 55 million older adults worldwide and presenting a significant burden on healthcare systems.^1^ AD and Alzheimer Disease and Related Dementias (AD/ADRD) encompass the neurodegenerative conditions that are characterized by progressive cognitive decline, functional impairment, and behavioral changes.^2^, ^3^, ^4^, ^5^, ^6^ The number of people with dementia is projected to reach 78 million in 2030 and 139 million in 2050, representing 10 million new cases of dementia each year worldwide.^1^ With this significant increase in dementia prevalence as populations age, addressing AD/ADRD has become a priority within public health agendas globally. The increasing prevalence of ADRD presents substantial socioeconomic and healthcare burdens, affecting individuals, families, and healthcare systems alike. Significantly, ADRD not only affects cognitive functions but also leads to functional decline, loss of independence, and increased caregiving needs. Furthermore, ADRD is associated with significant healthcare costs, including medical care, long‐term care, and caregiving expenses.^7^ The current global annual cost estimation for dementia exceeds 1.3 trillion USD, with projections indicating a rise to 2.8 trillion USD by 2030.^1^

AD is defined by its pathology rather than its symptoms. The pathological characteristics of AD were first identified by Alois Alzheimer in 1906.^8^ They include the presence of extracellular amyloid plaques comprised of amyloid‐β peptide and intracellular neurofibrillary tangles comprised of the microtubule‐associated protein tau.^5^, ^9^ Amyloid plaques and tau tangles are often associated with synaptic dysfunction, synaptic loss, and neurodegeneration. Biomarkers that reflect amyloid and tau pathology, therefore, enable clinicians to know whether AD pathology is present and may be causing or contributing to cognitive impairment. A more accurate diagnosis may enable better‐informed clinician, patient, and caregiver decisions.

However, timely and accurate clinical diagnosis of dementia remains challenging, especially in its early stages when interventions could be most effective.^10^, ^11^, ^12^ Current diagnostic methods are typically based on clinical evaluation and structural neuroimaging, which may lack sensitivity and specificity, especially in the early stages of the disease.^10^, ^13^, ^14^ Cerebrospinal fluid (CSF) biomarkers and amyloid positron emission tomography (PET) are available that can detect AD brain pathology and thereby assist clinicians in determining whether AD is a possible cause of cognitive impairment. However, the use of CSF biomarkers and amyloid PET in dementia diagnosis has been limited for multiple reasons.^15^ These modalities are restricted to tertiary medical centers, which only a small fraction of the US population uses. CSF collection via lumbar puncture (LP) is often perceived as invasive and risky, requires an experienced LP team, and is contraindicated in some patients, such as those on anticoagulation. PET imaging can be costly, which can be prohibitive for widespread use, particularly in resource‐limited settings. PET scans also subject patients to radiation exposure. Overall, the drawbacks of CSF biomarkers and PET have greatly limited their use in clinical dementia diagnosis.

In recent years, significant progress has been made in developing blood tests for AD. These blood tests include analytes measured in the blood serum or, more commonly, the blood plasma; the treatment of the blood sample determines whether serum or plasma is obtained. Biomarkers of amyloid and tau pathology include amyloid‐β (Aβ), especially the Aβ42 to Aβ40 ratio and tau phosphorylated at positions 181, 217, and 231 (p‐tau181, p‐tau217, and p‐tau231). It is important to note that while p‐tau biomarkers have traditionally been thought of as representing tau neurofibrillary tangles, they typically have even more robust associations with amyloid pathology and may represent a response to amyloid pathology. Neurofilament light chain (NfL) is a biomarker of neuroaxonal injury and neurodegeneration, and glial fibrillary acidic protein (GFAP) is a biomarker of astrocyte activation.

Blood tests offer advantages over traditional CSF and imaging biomarkers, such as relative non‐invasiveness, accessibility, and cost‐effectiveness.^10^, ^16^, ^17^ Blood tests are now widely used in research studies and clinical trials and are becoming more available for clinical diagnosis.^18^, ^19^, ^20^, ^21^ Integrating AD blood tests into clinical practice can revolutionize AD diagnosis and management by significantly reducing the time and cost involved in determining whether patients have AD pathology and may be eligible for AD‐specific treatments and clinical trials of experimental anti‐AD.^22^, ^23^ Moreover, blood tests have the potential to provide an earlier and more accurate diagnosis of dementia, allowing for personalized approaches to care and resource allocation.^17^, ^24^, ^25^

This review examines the current research status concerning AD blood tests, emphasizing their diagnostic, prognostic, and disease progression monitoring capabilities. With a specific focus on developments within the last 5 years, recent advancements in blood tests can potentially improve early detection, intervention, and research in AD, potentially revolutionizing dementia research and care.

## 2 METHODS

We conducted a comprehensive search of databases including PubMed, Embase, and Google Scholar using specific search terms: “Alzheimer disease,” “Alzheimer disease and related dementia,” AND “plasma,” OR “blood” OR “serum” AND “biomarkers.” Additionally, we reviewed reference lists to identify relevant articles not captured in the initial search. Our inclusion criteria prioritized articles published in English and focused on plasma biomarkers in AD in the last 5 years.

## 3 DISCUSSION

### 3.1 CSF biomarkers of AD

CSF and imaging biomarkers have been instrumental in understanding AD pathophysiology. Breakthroughs in detecting AD pathology via CSF biomarkers and imaging modalities represented significant milestones in the field of Alzheimer research.^25^, ^26^ The use of CSF biomarkers for AD gained prominence with the work of Trey Sunderland and colleagues. They found that CSF concentrations of Aβ42 and tau were associated with AD pathology.^27^, ^28^, ^29^, ^30^, ^31^ These CSF biomarkers have become essential for diagnosing AD in research and clinical settings.

Initially, CSF biomarkers for AD, such as Aβ42, total tau, and p‐tau181, were primarily used in research settings.^32^, ^33^ However, with advancements in technology and a growing understanding of AD pathology, several companies developed assays for detecting these biomarkers in CSF. There are now three assays for AD pathology that are approved by the United States Food and Drug Administration (FDA): the Lumipulse CSF Aβ42/Aβ40 test, and the Roche Elecsys p‐tau181/Aβ42 and total tau181/Aβ42 tests.^34^, ^35^, ^36^, ^37^ Appropriate use recommendations for CSF biomarkers have been described.^38^

### 3.2 PET biomarkers of AD

In 2004, William Klunk and Chester Mathis developed a groundbreaking imaging technique using a thioflavin derivative termed Pittsburgh Compound‐B (PiB) as a PET tracer.^39^, ^40^, ^41^ PiB binds to amyloid plaques in the brain, allowing researchers to visualize and quantify the extent of amyloid deposition.^40^

The FDA has approved various radioligands for clinical use, providing clinicians with valuable tools for detecting amyloid pathology in living individuals. The US FDA and the European Medicines Agency (EMA) have approved three radioligands, 18F‐florbetapir, 18F‐flutemetamol, and 18F‐florbetaben, for amyloid PET imaging, enabling visualization of amyloid plaques in cognitively impaired individuals evaluated for AD and other causes of cognitive decline.^42^, ^43^, ^44^, ^45^

### 3.3 Blood tests of AD

The FDA has not yet approved any blood tests for AD, but an increasing number are clinically available as laboratory‐developed tests. Importantly, there is a wide variation in the performance and validation of these tests. The first clinically available test for AD was PrecivityAD, offered by C2N Diagnostics in St. Louis, Missouri, USA, which used plasma Aβ42/Aβ40, age, and apolipoprotein E prototype to provide a risk score for amyloid PET positivity.^46^, ^47^, ^48^ Additional blood tests for AD include a plasma Aβ42/Aβ40 test offered by Quest Diagnostics and a plasma Aβ42/Aβ40, p‐tau181, and NfL test offered by Labcorp. More recently, the next generation of more accurate AD blood test based on p‐tau217 have become available. The PrecivityAD2® blood test offered by C2N Diagnostics uses plasma Aβ42/Aβ40 and the ratio of phosphorylated to non‐phosphorylated tau at position 217 to provide a likelihood of amyloid PET positivity.^49^ A plasma p‐tau217 test by ALZpath is also clinically available.^50^

### 3.4 Unique benefits of blood tests

Blood tests for AD offer several unique benefits compared to CSF analysis or PET imaging. The key advantages include accessibility, acceptability, cost‐effectiveness, repetitive monitoring, and potential for population screening.^16^, ^21^, ^51^ Blood samples can be easily collected in a clinical setting or at home, making it convenient for patients, especially those in remote areas or with limited mobility. This accessibility allows for broader screening and early AD detection, leading to timely interventions and improved outcomes. Blood tests are generally more acceptable to patients than LP, which is required for CSF collection. The non‐invasive nature of blood tests reduces patient discomfort and anxiety, leading to greater acceptance. This is particularly beneficial for older adults or individuals with cognitive impairment who may have difficulty with more complex procedures. In addition, blood tests for AD have the potential to be more cost‐effective compared to CSF analysis or imaging tests.^52^ The more straightforward sample collection process and lower infrastructure requirements reduce procedural costs. Additionally, the widespread availability of blood testing facilities further contributes to cost savings. As a result, blood tests for AD may offer a more affordable option for screening and diagnosing the disease.^23^, ^51^ Additionally, blood tests can be performed multiple times without substantial difficulty, allowing for repetitive monitoring of AD biomarkers over time. This longitudinal assessment enables clinicians to track disease progression, evaluate treatment efficacy, and adjust management strategies. Regular monitoring of blood tests may also facilitate early detection of changes associated with AD pathology, enabling timely interventions to slow disease progression.^53^

A significant advantage of blood tests for AD is their potential for widespread population screening.^54^ This accessibility could facilitate early detection and intervention strategies for AD. This research underscores the potential for these biomarkers to be used in blood tests, reflecting treatment response and disease progression. Blood tests offer a promising solution for population‐wide screening initiatives aimed at identifying individuals at risk of AD due to their accessibility, acceptability, and cost‐effectiveness. Routine screening using blood tests could potentially enable early intervention and preventive measures, potentially reducing the burden of AD. This could potentially enhance patient outcomes and alleviate the overall burden of AD in the broader population.

### 3.5 Potential challenges of blood tests

While blood tests for AD offer a non‐invasive and accessible diagnostic option, their reliability and accuracy are under scrutiny, with varying performance levels across tests.^55^ Factors such as age, sex, genetics, comorbidities, medications, and race/ethnicity can influence blood biomarker levels, potentially affecting diagnostic accuracy.^55^, ^56^, ^57^, ^58^, ^59^, ^60^, ^61^ A study by researchers at Washington University School of Medicine in St. Louis has shown that the race of people given AD blood tests may affect the interpretation of results.^62^ Three experimental blood tests used to identify people in the early stages of AD performed differently in Black individuals compared to White individuals, underscoring the need for reliable and accessible blood tests for AD. Additionally, in the United States, the costs of AD blood tests currently are not covered by insurance or reimbursed. Another significant limitation of the status of blood biomarkers is the lack of diversity in the research populations in which they have been evaluated, as the data have been derived from studies involving primarily White participants. This lack of representation means we know very little about the performance and reliability of these biomarkers in underrepresented groups (URGs). Addressing the variability and mitigating influencing factors in all populations will be essential for their widespread adoption and integration into clinical practice.^56^, ^57^ Challenges remain in translating blood tests from research settings to routine clinical practice. Standardization of assay methods, validation across diverse populations, and integration into existing diagnostic algorithms are critical steps in realizing the full potential of plasma biomarkers for AD.

### 3.6 Latest advancement in AD blood tests: State of the field

The field of blood tests for AD is rapidly evolving, with significant progress being made in recent years. Over the last 5 years, significant advancements have been made in developing blood tests for AD.^6^, ^21^, ^50^ Technological advancements have played a crucial role in developing plasma biomarkers for AD. Recent advancements in analytical techniques, including mass spectrometry, immunoassays, and multiplex assays, facilitate sensitive and accurate quantification of biomarkers in blood samples, thereby improving the detection of subtle changes linked to AD pathology.^63^, ^64^, ^65^, ^66^ Machine learning and artificial intelligence (AI) techniques have been increasingly used to analyze complex plasma biomarker study datasets.^66^, ^67^, ^68^ These methods help identify patterns, biomarker combinations, and predictive models that can improve the accuracy of AD diagnosis and prognosis based on blood test profiles.^68^ Machine learning algorithms can also aid in identifying individuals at risk of developing AD before clinical symptoms manifest.^69^, ^70^

Blood tests have emerged as valuable surrogate endpoints in clinical trials for evaluating the efficacy of potential AD therapeutics.^71^, ^72^ For example, a study by Salloway et al.^73^ highlighted the impact of gantenerumab and solanezumab on amyloid plaques using biomarkers. Additionally, a recent study by Wagemann et al.^74^ underscored the potential of blood tests to reflect changes in brain pathology and treatment responses in AD. The study highlighted critical vital blood biomarkers, such as GFAP and neurogranin, which are crucial for non‐invasive diagnostics. Observations included changes in GFAP and YKL‐40 levels in response to gantenerumab treatment, indicating these markers' utility in monitoring therapeutic responses. This support using blood tests as cost‐effective and accessible alternatives to CSF tests and neuroimaging, paving the way for personalized treatment strategies and improved patient care.

AD blood tests offer the potential for non‐invasive, cost‐effective, and easily accessible methods for diagnosing and monitoring AD pathology.^17^ Large‐scale validation studies help establish blood test's reliability, sensitivity, and specificity across diverse populations. Regulatory agencies, including the FDA, are increasingly interested in validating blood tests for AD diagnosis and patient stratification in clinical settings.^75^ Achieving regulatory approval requires rigorous validation and standardization of assays to ensure consistency and reproducibility.^5^

### 3.7 Context of use for AD blood tests

#### 3.7.1 Research studies

AD blood tests are becoming integral to various research and clinical endeavors, from understanding disease mechanisms to diagnosis, monitoring, and treatment. They offer insights into molecular changes associated with AD, aiding in early detection, risk assessment, and differentiation from other dementias.^17^ Longitudinal studies employing these biomarkers allow for disease monitoring and evaluation of intervention efficacy. Furthermore, they are pivotal in participant screening to determine eligibility for AD clinical trials and to inform population‐based studies, contributing to public health strategies. Additionally, blood tests may guide healthcare decisions, optimizing resource allocation and improving healthcare efficiency. Blood tests' versatile applications underscore the importance of ongoing research and innovation to fully harness their potential in enhancing outcomes for individuals with AD and their families.

#### 3.7.2 Clinical trials

Clinical trials leverage blood tests of AD for participant selection, treatment effect assessment, response prediction, and complication identification. Biomarker screening ensures appropriate participant selection by detecting AD pathology, aiding in selecting individuals for symptomatic and prevention trials. For instance, elevated amyloid‐beta and tau levels indicate AD pathology in those with cognitive impairment, guiding trial inclusion. In prevention trials targeting cognitively intact individuals, biomarkers identify those at risk of future AD, facilitating interventions to delay onset. Blood tests evaluate treatment effects on disease pathology and levels, supplementing clinical outcomes and imaging data.^76^ They predict treatment response based on baseline levels or changes during trials, aiding treatment stratification.^77^ Additionally, blood tests forecast complications, enabling proactive intervention.^78^ Blood tests streamline participant selection, treatment assessment, response prediction, and complication identification in AD clinical trials, enhancing trial efficacy and intervention development.^17^, ^79^

#### 3.7.3 Clinical practice

Blood tests for AD have the potential to revolutionize diagnostic approaches and enhance patient care. Offering a non‐invasive alternative to CSF analysis or PET scans, AD blood tests aid in diagnosing AD in symptomatic patients with cognitive impairment and treatment decisions.^10^, ^23^ Primary care physicians (PCPs) or geriatricians, who may not routinely perform CSF analysis or PET scans, can use blood tests to screen patients for further evaluation by dementia specialists, optimizing resource allocation and ensuring timely referrals. Dementia specialists, like neurologists, leverage blood tests to diagnose AD and other neurodegenerative disorders, enhancing diagnostic accuracy and treatment selection. Biomarker‐based algorithms identify patients likely to benefit from available treatments. Biomarker assessments, such as measuring p‐tau217 or NfL, monitor disease progression and treatment response, guiding treatment adjustments.^11^ In the future, blood tests may screen asymptomatic individuals for AD, enabling early interventions to delay or prevent dementia onset, thus reducing disease burden and improving long‐term outcomes. Thus, blood tests offer valuable diagnostic and prognostic insights in clinical practice, fostering early detection, accurate diagnosis, and personalized management of AD, potentially transforming AD diagnosis, treatment, and prevention.^23^

#### 3.7.4 Future directions and conclusion

Despite the remarkable recent progress in blood tests for AD, several avenues for future research and development exist. These include refining assay techniques, longitudinal validation studies, and exploring novel biomarkers and multi‐modal approaches. Ultimately, integrating blood biomarkers into routine clinical practice holds immense potential for improving early detection, prognostication, and therapeutic interventions in AD/ADRD. The emergence of these biomarkers represents a paradigm shift in AD/ADRD research, offering new insights into disease pathogenesis and progression. While challenges remain, ongoing efforts to harness blood tests' diagnostic and prognostic potential hold promise for transforming the landscape of AD/ADRD diagnosis and treatment.

## CONFLICT OF INTEREST STATEMENT

Suzanne E Schindler has analyzed data provided by C2N Diagnostics to Washington University. She has served on a Scientific Advsiory Board for Eisai and recevied speaker fees from Eli Lilly. John C Morris is funded by NIH grants #P30AG066444; P01AG003991; P01AG026276; Neither Dr. Morris nor his family owns stock or has equity interest (outside of mututal funds or other externally directed accounts) in any pharmaceutical or biotechonology company.
